# Computational modelling predicts activity-dependent neuronal regulation by nitric oxide increases metabolic pathway activity

**DOI:** 10.1186/1471-2202-16-S1-P84

**Published:** 2015-12-18

**Authors:** Christophe B Michel, Sarah J Lucas, Ian D Forsythe, Bruce P Graham

**Affiliations:** 1 Computing Science and Mathematics, University of Stirling, Stirling, Scotland, FK9 4LA, UK; 2 Dept Cell Physiology & Pharmacology, University of Leicester, Leicester LE1 9HN, UK

## 

The neuromodulator nitric oxide (NO), in addition to regulating electrophysiological homeostasis, post-synaptic receptor activity, and neuronal functions through cyclic GMP, has recently been shown to modulate the metabolic pathway. NO down-regulates mitochondrial activity [1] and the subsequent increase in AMP facilitates the activation of the phosphofructokinase enzymatic reaction in the glycolytic pathway [2].

Given these results, we want to better understand the regulation of neuronal energy metabolism by NO. To do that, we have built a computational model of energy metabolism based on prior works, principally on a model elucidating the control system structures of neuronal metabolism [3] and another highlighting the metabolic role between neuronal activity and hemodynamics [4]. From the first we took the biochemical pathway model, i.e. glycolysis, mitochondrial activity and regulation by the *astrocyte to neuron lactate shuttle*. From the second we took a model of intracellular sodium concentration and pumping by Na/K-ATPase. We added the activity dependent glutamate cycle [5], driven by electrophysiological activity. We finally completed these models with the previously described modulation by NO.

We have new experimental recordings of post synaptic currents in principal cells of the mouse medial nucleus of the trapezoid body (MNTB) in response to high frequency presynaptic stimulation. To match our model to this data we assumed the neurotransmitter released during electrophysiological activity to be proportional to the post synaptic current amplitude and so our glutamate cycle synaptic model, described above, could be related to the recorded EPSC amplitudes. The full metabolism model was then calibrated to match actual voltage clamp MNTB recordings of synapse activity, in the control case and in the presence of 5 mM 2-deoxy-D-glucose, blocking glycolysis, allowing only mitochondrial activity.

Model outputs are compared between (1) the baseline (control) conditions, (2) following conditioning with evoked activity that increases NO levels, modifying individually glycolysis and mitochondrial activity, and (3) in full NO regulated conditions, in order to evaluate the individual and mixed metabolism dynamics. Results show that ATP production is slightly increased by NO upregulation of glycolysis (2.5%), significantly increased by mitochondrial inhibition by NO (12.3%) and further increased (15.6%) when both glycolysis and mitochondria are NO modulated.

This model will eventually be combined with our previous postsynaptic model that shows NO modulation can reduce the cost of action potential generation in MNTB neurons [6] to build a more complete model of energy consumption during synaptic transmission.
